# Electrocardiographic safety evaluation of extended artemether-lumefantrine treatment in patients with uncomplicated *Plasmodium falciparum* malaria in Bagamoyo District, Tanzania

**DOI:** 10.1186/s12936-020-03309-2

**Published:** 2020-07-14

**Authors:** Lwidiko E. Mhamilawa, Sven Wikström, Bruno P. Mmbando, Billy Ngasala, Andreas Mårtensson

**Affiliations:** 1 Department of Women’s and Children’s Health, International Maternal and Child Health (IMCH), Uppsala University, Uppsala, Sweden; 2 Department of Parasitology and Medical Entomology, Muhimbili University of Health and Allied Sciences, Dar es Salaam, Tanzania; 3 Tanga Centre, National Institute for Medical Research, Tanga, Tanzania

**Keywords:** Malaria, *Plasmodium falciparum*, Cardiotoxicity, Artemether–lumefantrine, Tanzania, Prolonged treatment, Artemisinin resistance, ECG

## Abstract

**Background:**

Extended artemisinin-based combination therapy (ACT) for treatment of uncomplicated *Plasmodium falciparum* malaria with already existing drug regimens, such as artemether-lumefantrine, might be effective in tackling the emerging ACT resistance. However, given the history of cardiotoxicity among anti-malarial drugs structurally similar to lumefantrine, the potential effect of extended artemether-lumefantrine treatment on the electrocardiographic (ECG) QTc interval is of high concern.

**Methods:**

Male and non-pregnant females aged 1–65 years, diagnosed with uncomplicated *P. falciparum* malaria in Bagamoyo district, Tanzania, were randomized into two arms. The intervention arm received an extended, i.e. 6-day, course of artemether-lumefantrine and an additional single low-dose primaquine (0.25 mg/kg) administered together with the last artemether-lumefantrine dose. The control arm received the standard weight-based 3-day course. ECGs were performed at day 0 and 4–5 h after the last dose at day 5. QT intervals were read manually using the tangent method and automatically. Bazett’s (QTcB) and Fridericia’s (QTcF) formulae were used for correction for heart rate. Descriptive statistics were used to calculate baseline characteristics and the number of supra-thresholds QTc intervals (QTc prolongation > 500, change in QTc interval (ΔQTc) > 60 ms). The mean change in QTc interval in and between the two arms was compared using the paired t-test and independent samples t-test, respectively.

**Results:**

A total of 195 patients were enrolled, 103 and 92 in the intervention and control arm, respectively. No patient experienced QTc intervals > 500 ms on day 5 by both formulae. Patients with ΔQTc > 60 ms, for QTcF were 6/103 (5.8%) vs 2/92 (2.2%) and for QTcB 2/103 (1.9%) vs 1/92 (1.1%) in the intervention and control arms, respectively. The mean difference in ΔQTc interval was statistically significant between the two arms with both correction formulae, 11.4 ms (95% CI 2.7–20.0, p = 0.010) and 13.4 ms (95% CI 5.3–21.5, p = 0.001), for QTcB and QTcF, respectively.

**Conclusion:**

The extended 6-day course of artemether-lumefantrine did not reveal clinically relevant QTc prolonging effects. However, significant QTcF prolongation and presence of patients with supra-threshold QTc values observed in the intervention arm underscore the importance of further monitoring of QTc parameters in extended artemether-lumefantrine treatment.

*Trial registration* ClinicalTrials.gov, NCT03241901. Registered July 27, 2017. https://clinicaltrials.gov/show/NCT03241901

## Background

*Plasmodium falciparum* resistance to artemisinin-based combination therapy (ACT) is an emerging threat to the improvements made for past decade in the strive to control and eliminate malaria [1]. Alternative strategies to protect the therapeutic lifespan of artemisinin-based combinations are being explored, such as extending the ACT treatment duration, optimizing drug dosing, new combinations and use of triple ACT as development of new anti-malarial drugs with comparable efficacy is underway [2–6]. Among studies that explored the efficacy and safety of extended ACT, such as artemether-lumefantrine, i.e. the most commonly used artemisinin-based combination for the treatment of uncomplicated *P. falciparum* malaria in Africa, few have electrocardiographic (ECG) parameters as part of safety reporting [4, 6].

The QT interval in PQRST complex from an ECG, represents the time for ventricular depolarization and repolarization in the cardio-myocytes and it is correlated with the heart rate; the higher heart rate, the shorter QT interval and vice versa [7]. Because of this, the interpreted QT interval is corrected for the heart rate by various formulae to get a corrected QT interval (QTc). Commonly used formulae are Bazett’s (QTcB) and Fridericia’s (QTcF) among others [8, 9].

The importance of reporting the QTc interval from ECG in studies that involve anti-malarial drugs is anchored in the history of cardiotoxicity among some quinoline anti-malarial and structurally-related medicines [10]. Lumefantrine, the long-acting partner drug in artemether-lumefantrine, has chemical and structural similarities with halofantrine. Halofantrine has curtailed use as an anti-malarial drug since it is known for causing QTc prolongation and sudden cardiac death [11–13]. Studies of anti-malarial drugs that contain quinolines or structurally similar partner drugs have often included prolongation of the QTc as part of ECG evaluations. Prolongation of the QTc interval is a sensitive indicator for the risk of developing serious cardiac arrhythmias like Torsade de Pointes (TdP) and sudden death, albeit not highly specific [14, 15].

To date there has been no evidence that artemether-lumefantrine has caused serious cardiac adverse events in malaria patients when given as the standard weight based treatment course, i.e. twice daily, for 3 days (one tablet containing 20 mg artemether and 120 mg lumefantrine) [16–18], or as a single dose in healthy study participants [11]. However, a significant positive relationship between lumefantrine concentration and corrected QT interval by Fridericia’s (QTcF) was observed when healthy adults received a standard 6-dose regimen of artemether-lumefantrine over 3 days, in a trial performed by Novartis prior to seeking market approval [19]. Also, a recent clinical trial in Congo reported a statistically significant correlation between the QTcF interval, and the concentration of lumefantrine. The Congo study evaluated the efficacy, safety and tolerability of extending treatment with artemether-lumefantrine for 5 days among pregnant and non-pregnant women with uncomplicated *P. falciparum* malaria [6]. The extended treatment was found to be safe in this study population, and a maximum QTcF interval prolongation of 7.02–8.19 ms was seen. However, in the same study, corrected QT interval by Bazett’s (QTcB) did not demonstrate the correlation.

The Bazett’s formula is considered to be inferior to Fridericia’s in adult populations [20], however, consensus is lacking on which formula is best suited for reporting in paediatric populations [21, 22]. This is reflected in the heterogeneity of measurement methods and correction formulae that have been reported in studies of anti-malarial drugs, including customising study specific QT assessment methods which are independent of heart rate [14, 23]. The importance of continued monitoring of QTc prolonging effects for the new regimens of anti-malarial drugs and further evaluation of optimal methods for measuring and reporting the QTc interval should not be underestimated, given the history of cardiotoxicity among anti-malarial drugs.

This study investigated the effects of an extended course (6 days) of artemether-lumefantrine on QTc interval vs the standard (3 days) course in male and non-pregnant female patients with uncomplicated *P. falciparum* malaria in Bagamoyo District, Tanzania, and compared the outcomes between QTcB and QTcF.

## Methods

### Study site and population

This study was part of a clinical trial with the aim of comparing extended 6 days of artemether-lumefantrine vs standard treatment i.e. 3 days course on PCR determined parasite clearance, cure rate, post-treatment prophylaxis, and safety and tolerability. Full description and results from the parent study will be reported in a separate publication, but briefly; 280 patients with microscopy confirmed uncomplicated *P. falciparum* malaria were enrolled in Yombo and Fukayosi dispensaries, Bagamoyo District, Tanzania, after giving written informed consent. The study was conducted in accordance with the Declaration of Helsinki and Good Clinical Practice, and was granted ethical approval from the National Institute for Medical Research (NIMR/HQ/R.8a/Vol.IX/2477) and Muhimbili University of Health and Allied Sciences, Tanzania, (MU/DRP/ERP/Vol.IX/174) and the Regional Ethical Review Board, Stockholm, Sweden. The study is registered on https://www.clinicaltrials.gov (NCT03241901).

### Inclusion criteria

Male and non-pregnant female patients were included if they were between 1 and 65 years old, had a weight of 10 kg and above, body temperature ≥ 37.5 °C or history of fever within the last 24 h, microscopy determined asexual *P. falciparum* mono-infection (regardless of parasitaemia) and a QTcB interval at baseline ECG < 440 ms in males and < 460 ms in females. The QTcB interval used for enrolment was automatically measured value from the ECG machine.

### Exclusion criteria

Patients were excluded from the study in case of any symptoms/signs of severe malaria or other danger signs, pregnancy, breastfeeding or if the patient was unwilling to practice birth control during participation in the study, if they had a known allergy to study medications or known cardiac disorders, Hb < 8 g/dl, reported anti-malarial intake within 2 weeks, regular medication which may interfere with anti-malarial pharmacokinetics or had received a blood transfusion within the last 90 days.

### Study design

The patients were randomized into control and intervention arms in this open, single-blinded trial. The patients in the control arm received artemether-lumefantrine tablets (20/120 mg) (Coartem^®^, Novartis Pharma, Switzerland) in accordance with their body weight and the Tanzanian national treatment guidelines for uncomplicated *P. falciparum* malaria, i.e. twice a day for 3 consecutive days, as follows: one tablet to patients weighing 5–14 kg; two tablets to children weighing 15–24 kg; three tablets to children weighing 25–34 kg and four tablets for patients with a weight above 35 kg. In the intervention arm, patients received artemether-lumefantrine according to the protocol described above for 6 consecutive days. In addition to this, a single dose of 0.25 mg/kg primaquine (Primaquine phosphate, Sanofi) was given together with the last dose of artemether-lumefantrine as supported by modelling data, which suggest that a later primaquine dose has improved gametocidal effect compared to giving with first dose [24]. ECGs were performed by a qualified physician for both treatment arms, at two time points, i.e. before enrolment and 4–5 h after the 12th (final) dose of artemether-lumefantrine in the extended treatment arm and at the 12th visit (3 days after the last dose) for the control arm.

### QT measurement method and QT correction for heart rate

Standard 12–lead ECGs were recorded at speed of 25 mm/s and amplitude of 10 mV/mm with two different machines, one from each study site, the Sonoscape ECG IE12 (Shenzhen, China) and CardiMax FX–7402 (Fukuda Denshi USA). The interval between one R wave and the next (RR intervals) in an ECG, were derived from the heart rate values, which were measured automatically by the machines.

In addition to the automatically obtained values, manual measurement using the tangent method was performed by a single analyst blinded to treatment arm. Prominent U-waves (≥ 50% of the height of the T-wave) were not included in QT interval, neither were discrete U-waves. Four different values were measured in lead II with a precision of 0.5 mm. If not readable in lead II, the QT interval was measured in the chest leads, preferably V1–V2. The mean of the four QT interval values was taken as final QT interval, then corrected for heart rate with Bazett’s (QTcB = QT/RR^1/2^) and Fridericia’s (QTcF = QT/RR^1/3^) formulae, respectively [25, 26]. An additional age adjusted QTc interval was generated by combining QTcB of children < 10 years and QTcF of patients ≥ 10 years to get a “QTc-age”.

Cut-off values of > 500 ms for QTc prolongation or > 60 ms for change in QTc (ΔQTc) interval values from baseline were used to categorize supra-thresholds QTc intervals i.e., QTc intervals exceeding thresholds of clinical concern [27, 28].

### Other safety parameters

Haemoglobin concentration was measured during enrolment and at day 7, using a portable spectrophotometer, HemoCue Hb 201 + (HemoCue AB, Ängelholm Sweden). Venous blood (2 ml) was collected during enrolment and at day 7, to asses liver integrity by alanine aminotransferase (ALAT), aspartate aminotransferase (ASAT), serum bilirubin levels and kidney integrity by creatinine levels. The samples were stored for a maximum of 48 h in the field refrigerator (4 °C) before transport to an ISO certified reference laboratory at the Bagamoyo Research and Training Unit (Ifakara Health Institute) for analysis. The biochemistry analyses were done using COBAS INTEGRA 400 plus (COBAS, USA). The values were compared with age specific normal ranges [29].

### Outcomes

The primary outcomes of this study were to compare (1) the mean change in QTcB and QTcF interval between day 0 and day 5, and (2) the number of supra-thresholds QTc intervals (i.e. QTc > 500 ms or ΔQTc > 60 ms), within and between the arms. Secondary outcomes included investigating the number of QTcB and QTcF intervals exceeding 480 ms, and comparing the performance of Bazett’s and Fredericia’s formulae in correcting the QT for heart rate.

### Statistical analysis

Descriptive statistics were used to summarize weight, age, heart rate and body temperature for the patients at baseline and supra-thresholds QTc intervals. Changes in mean QTc interval from baseline and the last measurement for both correction formulae and QTc-age were compared by using *paired t*-*test*. Mean changes in QTc interval between day 0 and 5 were compared between the treatment arms with the *independent sample t*-*test*. Bazett’s and Fridericia’s formulae were used in correction of heart rate. An attempt to assess the mean change in QTc independently from correction formulae and heart rate changes was done by analysing the intercepts of ΔQTc vs ΔRR regression. Changes in heart rates and body temperature between the two time-points were calculated with the *paired t*-*test*. QTc values for both correction formulae and QTc-age were plotted against the RR intervals superimposed with a line of best fit from a linear regression model. A p-value < 0.05 was considered statistically significant. All statistical analyses were made in IBM SPSS statistics (version 26.0. Armonk, NY: IBM Corp).

## Results

### Patient flow and demographic data

**Fig. 1:**
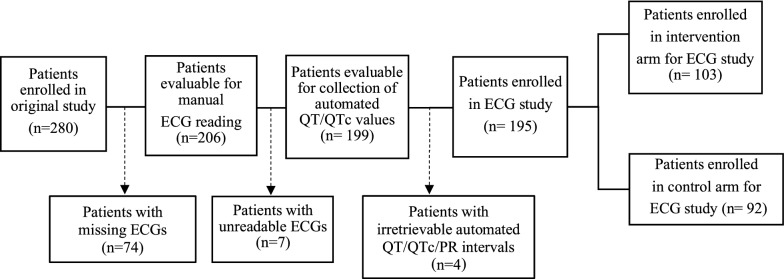
Flow chart of study participants

The flow of patients through the ECG safety evaluation study is presented in Fig. 1. A total of 195/280 patients were eventually included in the study. Baseline clinical and demographic characteristics were comparable between the treatment arms (Table 1). The mean age in the group of excluded patients was significantly lower (p = 0.047) than the mean age among the included patients (11.6 compared to 15.0 years). On average, heart rate decreased by 20.4 bpm between day 0 and day 5. There was no statistically significant difference between the intervention and control arm in mean change in heart rate between day 0 and day 5. Heart rate data are shown in Table 2.

**Table 1:** Baseline characteristics of included patients

	Intervention arm (n = 103)	Control arm (n = 92)	P value
Age (years)	11.0 (6.0–14.0)	12 (6.3–26.5)	0.284^†^
Sex, female; male	48; 55	37; 55	0.389^¥^
Weight (kg)	28.0 (20.0–45.0)	29.5 (20.3–54.3)	0.952^†^
Temperature (°C)	38.4 (37.7–39.2)	38.4 (37.7–39.1)	0.965^†^

Data are medians (interquartile range)

^†^Non parametric independent samples median test

^¥^Fisher’s exact test

**Table 2:** Heart rate data

	Intervention arm (n = 103)	Control arm (n = 92)	P value^†^
Mean heart rate day 0	105.5 (23.7)	101.7 (24.6)	0.708
Mean heart rate day 5	82.9 (16.0)	83.8 (17.0)	0.284
Mean change in heart rate between day 0 and 5	− 22.5 (18.8)	− 18.0 (19.3)	0.095
P value^¥^	*< 0.001*	*< 0.001*	

Heart rate values were measured in BPM (beats per minute). Data are means (standard deviation)

Italic values indicate significance of p value (p < 0.05)

^†^P value was compared using the independent t*-*test for difference in the mean change in heart rate and mean heart rate between the treatment arms in respective time*-*points

^¥^P value was compared using paired t-test for difference in mean heart rate within the treatment arms between day 0 and 5

There was no statistically significant difference in body temperature or mean change in body temperature between the two arms. A statistically significant decrease of 1.7 °C and 1.6 °C was seen in the intervention and control arm, respectively. Serial body temperature data are presented in Table 3.

**Table 3:** Body temperature data

	Intervention arm (n = 103)	Control arm (n = 92)	P value^†^
Mean temperature day 0	38.4 (1.0)	38.3 (1.0)	0.542
Mean temperature day 5	36.7 (0.3)	36.7 (0.3)	0.474
Mean change in temperature between day 0 and 5	− 1.7 (1.1)	− 1.6 (1.0)	0.419
P value^¥^	*< 0.001*	*< 0.001*	

Body temperature values were measured in °C (degrees Celsius). Data are means (standard deviation)

Italic values indicate significance of p value (p < 0.05)

^†^P values were compared using the independent t*-*test for difference in the mean change in body temperature and mean body temperature between the treatment arms in respective time*-*points

^¥^P values were compared using paired t*-*test for difference in mean body temperature within the treatment arms between day 0 and 5

### QTc data

Compared to the baseline, there was a statistically significant increase of the mean QTcF interval by 16.2 ms (p < 0.001) in day 5 in the intervention arm, while there was no significant difference in the same period when using Bazett’s formula in the intervention arm. In the control arm, a significant change (p = 0.004) was observed when correction by Bazett’s was used (Table 4). Generally, the QTc values obtained with Bazett’s correction formula were higher than the intervals corrected for heart rate with Fridericia’s formula.

**Table 4:** ECG data

Arm	Correction formula	Day	QTc (ms)	Absolute QTc prolongation		Change from baseline		
			Mean QTc (SD)	QTc > 480 ms	QTc > 500 ms	ΔQTc > 60 ms	Mean (95% CI)	P value*
Intervention (n = 103)	Bazett’s	0	410.2 (25.4)	1	1	1	1.9 (− 3.9–7.8)	0.513
		5	412.1 (25.0)	0	0			
	Fridericia’s	0	375.3 (26.0)	0	0	6	16.2 (10.6–21.7)	*< 0.001*
		5	391.5 (21.2)	0	0			
	QTc-age	0	393.4 (31.2)	0	0	4	9.1 (3.2–15.0)	*0.003*
		5	402.5 (25.7)	0	0			
Control (n = 92)	Bazett’s	0	416.8 (32.1)	4	1	1	− 9.4 (− 15.8–(− 3.0)	*0.004*
		5	407.4 (27.5)	1	0			
	Fridericia’s	0	383.6 (30.2)	1	0	2	2.7 (− 3.3–8.8)	0.371
		5	386.3 (23.4)	0	0			
	QTc-age	0	400.9 (35.2)	2	1	1	− 3.0 (− 9.7–3.6)	0.367
		5	397.9 (27.7)	0	0			

Italic values indicate significance of p value (p < 0.05)

* P values were calculated using the paired t-test for change in mean QTc interval between day 0 and 5 in each treatment arm with Bazett’s and Fridericia’s formulae and age adjusted QTc values (QTc-age), respectively. All QT intervals were manually derived

### Comparison of mean QTc change between treatment arms

There was a statistically significant difference in mean QTc change between the intervention and control arm with both correction formulae, 11.4 ms for QTcB and 13.4 for QTcF, respectively (Table 5). The difference existed also when analysing the automatically obtained data, with a mean QTcB change of 9.5 ms (p = 0.006). Table 6 presents resuts of comparing intercept of the ΔQTc vs ΔRR as mean (SD) of each arm for different QT correction formulae, which was statistically significant different between arms. There was no statistically significant difference between the manually and automatically obtained data, when comparing mean QTcB change in all patients.

**Table 5:** Mean QTc change between treatment arms

	Intervention arm (n = 103)	Control arm (n = 92)	Mean difference	95% CI	P value*	All patients (n = 195)
ΔQTcB^ψ^	1.9 (30.1)	− 9.4 (30.9)	11.4	2.7–20.0	*0.010*	− 3.4 (30.9)
ΔQTcF^ψ^	16.2 (28.2)	2.7 (29.1)	13.4	5.3–21.5	*0.001*	9.8 (29.3)
ΔQTcB^¥^	3.1 (22.1)	− 6.4 (25.6)	9.5	2.7–16.2	*0.006*	− 1.4 (24.2)

Data are means (standard deviation). Values are reported in milliseconds

Italic values indicate significance of p value (p < 0.05)

* P values were calculated using the independent t-test for difference in mean change in QTc interval between the treatment arms

^ψ^Manually measured data

^¥^Automatically obtained data from machine

**Table 6:** Comparing intercept of the ΔQTc vs ΔRR as mean (SD) between arms for each formulae

Correction formulae	Arm	Mean (SD)	95% CI	P-value*
Bazett’s	Intervention	10.6 (38.6)	3.2–18.1	*0.014*
	Control	− 2.4 (35.0)	− 9.6–4.8	
Fridericia’s	Intervention	11.1 (36.5)	3.9–18.3	*0.014*
	Control	− 1.3 (33.6)	− 8.2–5.6	
QTc-age	Intervention	10.5 (38.4)	3.0–18	*0.022*
	Control	− 1.9 (36.5)	− 9.5–5.7	

Italic values indicate significance of p value (p < 0.05)

* P values were calculated using the t-test for comparing the means of mean change in QTc interval between day 0 and 5 in each treatment arm

In terms of supra-threshold intervals, no QTc interval exceeded 500 ms in either of the arms with Fridericia’s formula. However, one patient from each arm had QTcB interval exceeding this threshold. Importantly, in both cases this occurred prior to treatment initiation (day 0). One patient in the control arm exceeded the lower threshold (QTc > 480 ms) on day 5 when using Bazett’s formula, whilst no patient in the intervention arm did. With Fridericia’s formula 6/103 (5.8%) patients in the intervention arm had a QTc lengthening of more than 60 ms, as compared with 2/92 (2.2%) in the control arm. With Bazett’s formula, the number was 2/103 (1.9%) in the intervention arm and 1/92 (1.1%) in the control arm. Individual data on patients with a QTc prolongation of more than 60 ms are shown in Additional file 1: Table S1.

### QTc/RR regression

**Fig. 2:**
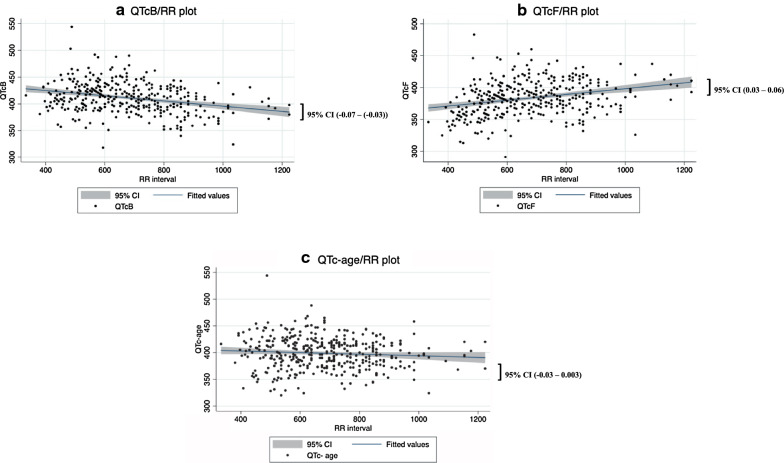
**a** QTcB/RR plot, **b** QTcF/RR plot, **c** QTc-age/RR plot superimposed with line of best fit from linear regression

QTc values were plotted against the RR intervals (Fig. 2). The plot shows that QTcB decreased as RR interval increased while QTcF increased as RR interval increased. The plot with QTc-age demonstrated an improved independence to RR interval with an R^2^ value of 0.007 of the regression lines. The slopes (95% CI) of the regression lines of QTcB, QTcF and QTc-age was − 0.049 (− 0.065–(− 0.033)), 0.046 (0.030–0.061) and − 0.015 (− 0.033–0.003), respectively.

## Discussion

### Safety

Previous studies have shown that WHO approved therapeutic doses of artemisinin-based combination can prolong the QTc interval in patients treated for uncomplicated *P. falciparum* malaria, but without causing arrhythmias or sudden deaths secondary to cardiac manifestations associated with ACT [15, 23]. In this study, prolonging treatment with artemether-lumefantrine for a total of 6 days, together with a single low dose of primaquine was found to be safe with no patients experiencing any cardiac adverse events. There were no patients in this study with QTc intervals > 500 ms on day 5, and only 6/103 (5.8%) (QTcF) and 2/103 (1.9%) (QTcB) patients had individual ΔQTc > 60 ms in the intervention arm. This proportion of patients with a ΔQTc > 60 ms, is comparable to previous results from studies of standard weight-based 3 days artemether-lumefantrine treatment in African children [17]. Interestingly, and similarly with these data, the amount of patients with a QTc change from baseline > 60 ms was higher with Fridericia’s formula compared to Bazett’s [17]. This might be explained by an inherent tendency of overcorrection at high heart rates and under correction at low heart rates that occurs with Bazett’s formula.

It is important to note that none of the patients had risk factors that would have predisposed them to prolonged QTc interval, such as renal impairment (that would have deranged the electrolytes), hepatic impairment, concomitant medication with proarrhythmic effects, or know pre-existing cardiac conditions [30]. Moreover, significant myocardial dysfunction and arrhythmias are rarely seen even in severe malaria cases, and it has been argued that electrocardiographic monitoring for patients with malaria infection may be of questionable clinical value if the purpose is not to study potential drug induced cardiotoxicity [31, 32]. However, one of the main findings of this study was the significant lengthening of the QTcF interval of 16.2 ms in the intervention arm (p < 0.001). This prolongation is comparable with some previous literature for artemether-lumefantrine [23], and it is even longer when compared to other literatures [6, 19, 33]. No statistically significant QTc prolongation was seen with Bazett’s formula in the same treatment arm.

The significant mean difference in mean QTc change between the two arms, 11.4 ms and 13.4 ms with Bazett’s and Fridericia’s formula, respectively, might be related to differences in lumefantrine exposure, where the intervention arm received twice the amount of lumefantrine compared with the control arm, and also at the time of measurement of QTc values in the control arm, it was 3 days past the last artemether lumefantrine dose. In the Novartis safety trial for artemether-lumefantrine given therapeutic doses, a model predicted lumefantrine Cmax of 480 mg correlated with prolongation in mean change in QTcF of 7.0 ms (90% CI, 5.5 –8.5) [19]. In this study the observed QTcF prolongation (16.2 ms) was almost as twice as the model predicted for Novartis therapeutic doses in the intervention arm who received double the lumefantrine exposure. Not having individual pharmacokinetics data in this study, limits the precision of the comparison.

Recently an extensive systematic review and meta-analysis of factors affecting QT interval in malaria patients and healthy individuals was published, involving more than 10,452 individuals (93.6% had microscopy confirmed *Plasmodium falciparum* or *Plasmodium vivax* infection). It provided compelling evidence of the contribution of malaria disease severity, changes in heart rate and body temperature in affecting the QT interval during recovery phase, where fever has an effect independent of heart rate. This brings to light the importance of taking into account disease process and other factors like age and sex when evaluating the effects of the anti-malarial drugs (quinolines) or other important medications on QT interval. By doing so, it may be possible to avoid unnecessary withdrawal of potent anti-malarial drugs currently used in malaria case management, or unnecessary discontinuation of anti-malarial drug development because of excessive attribution the QT prolonging effects of the drugs [34].

### Correction formulae

There was no significant difference between the manually and automatically obtained QTcB intervals, which together with the large number of study participants suggests that the estimated QT intervals are quite representative of the true QT intervals. Bazett’s formula can be used in similar studies for comparisons with historical data, but the QTcB intervals should probably be interpreted with caution compared to QTcF intervals due to its known limitations for adult populations. As expected, when QTc intervals were plotted against RR interval, there was an overcorrection at high heart rates and under-correction at low heart rates with Bazett’s formula (Fig. 2). The regression slopes were generally in line with previous results [35], although the QTcF slope reveals an under-correction at high heart rates and overcorrection at low heart rates. When study specific age adjustments were made (QTc-age) to analyse individual data, the effect of heart rate on QTc interval was reduced. The 95%CI of the slopes of regression lines differ from zero for Fridericia and Bazett regression but not for QTc-age. This provides a potentially useful method of presenting individual QTc data that is not influenced by the change in heart rate, reducing the limitations observed under the Bazett and Fridericia methods in this population with high range in patient ages.

Moreover, the analysis method of comparing the intercept of ΔQTc vs ΔRR regression between the groups as mean change of QTc that is independent of the differences between Bazett and Fridericia, provided relatively consistent results across the formulae (Table 6). This important finding is supportive of the robustness of the approach when it comes to harmonizing the formulae dependent discrepancies during analysis of QTc interval for mean (SD) change in QTc as was observed in Table 4. However, this methodology limits assessment of individual values of QTc. Generally results are in line with previous studies that recommended the approach of using study specific adjustments for appropriate estimations of QTc parameters [21, 22].

### Study limitations and potential confounders

With an ECG recording speed of 25 mm/s, as in this study, the reading precision might be lower than with a recording speed of 50 mm/s. Therefore, a reading precision of 0.5 mm was chosen. Also, cut-off thresholds were chosen with this in mind. Cut-off values differ between studies, but there’s consensus regarding some thresholds of absolute clinical concern, which were chosen for this study (QTc interval > 500 ms, ΔQTc > 60 ms) [28]. There has been concern regarding non QT experts’ ability to correctly identify a prolonged QTc interval [36]. However, the QT defining method used in this study (the tangent method) has been shown to be accurate when performed by non QT experts [37].

For a fully accurate comparison between the two arms to be made, ECG recordings obtained at the estimated peak drug concentration in the control arm (4–5 h after the last and 6th dose) would be of high value. Due to the setting of this sub study, and prior studies of the effects on QTc interval from the standard weight based 3-day course of artemether-lumefantrine [6], ECG recordings on day 2 were not performed. The absence of this data curbs the comparisons of absolute QTc interval prolongation between the two arms and constitutes an apparent limitation of this study. Also, in the intervention arm, potential drug–drug interaction due to the addition of single low-dose primaquine calls for cautious interpretation of the observed QTc prolongation. Primaquine is a quinoline which is structurally similar to lumefantrine.

Moreover, the disease process can be a potential confounder in the study. Patients with acute uncomplicated *P. falciparum* infection often experience fever and anxiety, which contribute in sympathetic activation and increased heart rate, leading to shorter RR interval and subsequent relatively shorter QTc interval. With appropriate treatment these symptoms decrease, and the QTc interval lengthens [10, 14, 38]. This poses a potential limitation when it comes to measuring potential QTc prolonging effects from therapeutic drugs, since no correction formula is totally independent of heart rate, and the changes in heart rate before and after treatment makes it more difficult to separate the drug effect on the QTc interval from the effects due to regression of symptoms [23].

The sample size for this sub study was calculated for primary outcomes of the parent study where overall safety assessment was done, but not primarily powered according to ECG parameters.

## Conclusion

The extended 6-day course of artemether-lumefantrine for patients diagnosed with uncomplicated *P. falciparum* malaria in Bagamoyo District, Tanzania, did not have a clinically relevant effect on the QTc interval. However, the significant QTcF prolongation and presence of patients with supra-threshold QTc values observed in the intervention arm underscore the importance of further monitoring of QTc parameters in extended artemether-lumefantrine treatment studies.

## Supplementary information

**Additional file 1: Table S1.** Patients with QTc prolongation > 60 ms from baseline.

## Data Availability

The datasets analysed during the current study are available from the corresponding author on reasonable request.

## Abbreviations

ACT: Artemisinin-based combination therapy
BPM: Beats per minute
CI: Confidence interval
ECG: Electrocardiogram
FDA: United States Food and Drug Administration
IQR: Inter quartile range
ICH: International Conference on Harmonisation of Technical Requirements for Registration of Pharmaceuticals for Human Use
Kg: kilograms
ms: milliseconds
MUHAS: Muhimbili University of Health and Allied Sciences
NIMR: National Institute for Medical Research
QTcB-interval: QT-interval corrected for heart rate with Bazett’s formula
QTcF-interval: QT-interval corrected for heart rate with Fridericia’s formula
QTc-interval: QT-interval corrected for heart rate
SD: Standard deviation
TdP: Torsade de Pointes
WHO: World Health Organization
