# Supplementary material for: Electrocardiographic safety evaluation of extended artemether-lumefantrine treatment in patients with uncomplicated Plasmodium falciparum malaria in Bagamoyo District, Tanzania
Source: Malar J. 2020 Jul 14;19:250. doi: 10.1186/s12936-020-03309-2 (PMC7362422; doi:10.1186/s12936-020-03309-2)
Supplement: Supplementary file 1 — Additional file 1: Table S1. Patients with QTc prolongation > 60 ms from baseline. [file 12936_2020_3309_MOESM1_ESM.docx]

| Sex, Age (years) | Arm | Weight  (kg) | Hb day 7 (g/dl) | ALAT day 7  (IU/L) | ASAT day 7  (IU/L) | Total  Billirubin  day 7 (µmol/L) | Creatinine day 7  (µmol /L) | Heart rate  (bpm) | QTcF  (ms) | QTcB  (ms) | ΔQTcF  (ms) | ΔQTcB  (ms) | Temp  (°C) |
| --- | --- | --- | --- | --- | --- | --- | --- | --- | --- | --- | --- | --- | --- |
| Female, 16 | I | 38 | 12.7 | 12.4 | 21.8 | 7 | 47 | 109  (75) | 329  (399) | 364  (414) | 70 | 50 | 39  (36.3) |
| Male, 4 | C | 12 | 12.2 | 16.8 | 26.6 | 6.3 | 18.9 | 136  (103) | 315  (377) | 361  (413) | 62 | 52 | 39.9  (37) |
| Female, 14 | I | 39 | 13.2 | 11.3 | 13.9 | 7.3 | 36.3 | 107  (79) | 352  (415) | 387  (436) | 63 | 49 | 38.7  (37.1) |
| Male, 13 | I | 32 | 12.7 | 10.0 | 15.5 | 4.3 | 34.8 | 137  (82) | 356  (422) | 408  (444) | 66 | 36 | 38.4  (37) |
| Female, 29 | C | 56 | 10.4 | 10 | 17.3 | 6.3 | 58.3 | 117  (85) | 331  (410) | 370  (434) | 79 | 64 | 38.4  (36.9) |
| Female, 5 | I | 19 | 10.9 | 23 | 23.5 | 3.3 | 22.36 | 122  (91) | 329  (391) | 371  (419) | 62 | 48 | 38.4  (36.8) |
| Female, 35 | I | 63 | 12 | 36.2 | 28.9 | 4.7 | 32.7 | 112  (84) | 320  (403) | 355  (426) | 83 | 71 | 39.9  (36.9 |
| Female, 9 | I | 25 | 11 | 13.3 | 16.1 | 3.8 | 32.6 | 102  (72) | 358  (430) | 391  (444) | 72 | 53 | 39  (37.1) |

**Table S1 – Patients with QTc prolongation >60ms from baseline**

The numbers reported are day 0 values (day 5).
